# Timescale mismatch: redefining ecological opportunity for evolution in the Anthropocene

**DOI:** 10.1038/s44318-026-00880-3

**Published:** 2026-07-31

**Authors:** Christian R Voolstra, Ruben J Hermann, Herwig Stibor, Alexander Keller, Lutz Becks

**Affiliations:** 1 Department of Biology, University of Konstanz, Konstanz, Germany; 2 Faculty of Biology, Ludwig-Maximilians-Universität München, Planegg-Martinsried, Germany

**Keywords:** Evolution & Ecology, Microbiology, Virology & Host Pathogen Interaction

## Abstract

This Commentary discusses how the rapid pace of environmental change in the Anthropocene redefines ecological opportunity.

**Figure:**
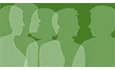

## Teaser

The Anthropocene is defined by unprecedented biodiversity loss, but also by a profound mismatch of timescales: environmental change unfolds orders of magnitude faster than response times for most ecological and evolutionary processes. Rapid perturbations create transient ecological opportunities, yet these windows are often too brief for diversification to manifest. The central challenge to understanding biodiversity change in the Anthropocene is not whether ecological opportunity—the availability of novel resources or niches that relax competition—arises, but whether it persists long enough for evolution to act. Microbiome-mediated as well as behavioral and physiological plasticity can buffer organisms against short-term instability and enable swift niche shifts, but such flexibility is reversible, typically non-heritable, and fragile under sustained disturbance. Without enduring selection or sufficient genetic variation, flexibility cannot substitute for evolution. The classical framework of ecological opportunity—conceived for stable environments and gradual change—is therefore inadequate. Diversification, i.e., the rebuilding of biodiversity in the Anthropocene, demands a time-scale integrated framework that unites evolutionary, ecological, and microbiome dynamics to pinpoint when fleeting opportunities can be stabilized for lasting diversification.

## Introduction

Biodiversity is not a fixed condition, but the time-integrated emergent outcome of evolutionary diversification, ecological interactions, and host–microbiome dynamics. For decades, ecological opportunity has provided a cornerstone for explaining how biodiversity arises: when unoccupied or underused portions of niche space become available, organisms expand their realized niches, and—if opportunity persists—evolutionary diversification follows. This logic has framed classic examples of adaptive radiation following island colonization, key innovations, or mass extinctions (Yoder et al, 2010; Wellborn and Langerhans, 2015; Schluter, 2000; Simpson, 1984; Gillespie, 2004). Yet the Anthropocene has rewritten the pace of change in ways that challenge this foundational paradigm.

## Timescale mismatch: redefining ecological opportunity

Environmental change now proceeds at unprecedented rates. Climate extremes can unfold within days, landscapes fragment within years, and species can disappear within only a few generations of the organisms concerned (Díaz et al, 2019; Rull, 2022; Barnosky et al, 2011; Hallmann et al, 2017; Exposito-Alonso et al, 2022; Newbold et al, 2018; Fischer et al, 2021). This compression of timescales creates a deep temporal mismatch: ecological processes and microbiome shifts occur rapidly, while evolutionary change remains constrained by generation time and genetic variation. Although microbiome shifts are ecological in origin, we distinguish them from broader ecological change because they can occur on particularly short timescales and provide temporally resolved signatures of rapid community reconfiguration, whereas most ecological processes operate continuously over longer periods. Whether biodiversity built-up is realized depends less on whether opportunity arises than on how long ecological and genetic processes—dispersal, admixture, and mutation—can sustain it before collapse. Ecological opportunity, once a protracted phase where selection could operate, has become transient and unstable. Niche space still opens—after wildfires (Burkle et al, 2015), floods (Lloren and McCune, 2024), or invasions by changing biotic interactions (Keane, 2002)—but often closes before populations respond.

Microbiome-mediated plasticity alongside behavioral and physiological plasticity appears to offer a bridge and may provide a rapid response layer. Microbial communities can restructure within days, and holobiont functional states can shift even more rapidly, due to metabolic plasticity and functional redundancy, enabling hosts to adjust to novel conditions (Fontaine et al, 2022; Ziegler et al, 2017; Xiang et al, 2022). Still, this adaptive flexibility is often temporary and easily disrupted by ongoing disturbance. However, recent work suggests that microbiome-mediated plastcity can, in certain cases, be transmitted to subsequent generations, as offspring may harbor specific members of acclimated microbiota (Baldassarre et al, 2022). Even when microbiome-associated states are inherited, their persistence depends on transmission fidelity, host filtering, and environmental reinforcement. Thus, rather than substituting for evolution, it dampens selective gradients, buffering stress and thereby short-circuiting genetic adaptation in many systems (Ghalambor et al, 2007; Fox et al, 2019).

The Anthropocene therefore demands redefining ecological opportunity as a fundamentally timescale-dependent phenomenon. Rather than assuming available ecological opportunity naturally leads to diversification, we must identify when—and for how long—transient openings persist amid accelerating change. Integrating evolution, ecology, and microbiome science across mismatched temporal domains reveals when short-lived windows become enduring arenas for selection as opposed to cases where plasticity merely delays collapse.

## Ecological opportunity and diversification in brief

**Figure. 1:**
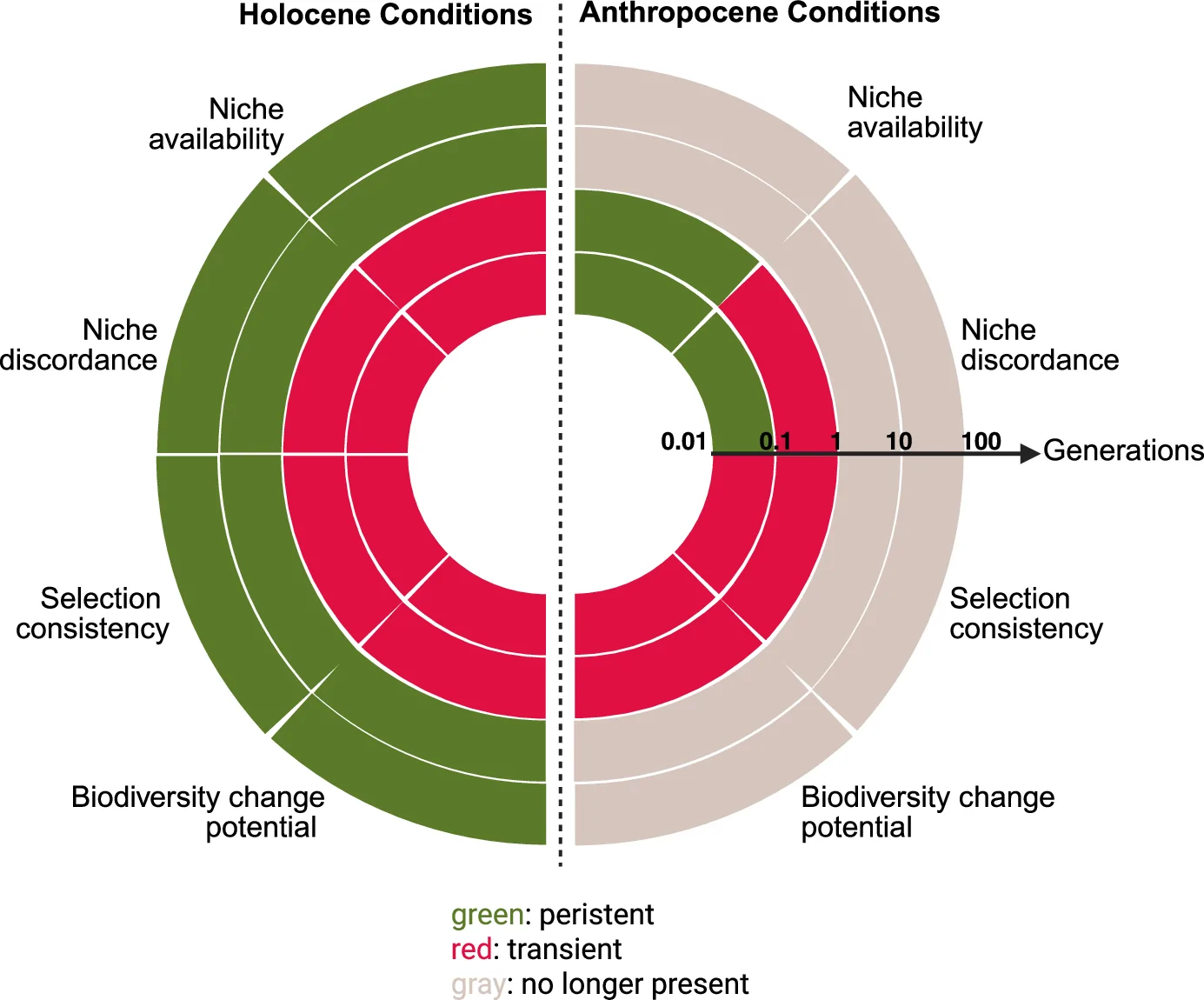
Timescale mismatch constrains ecological opportunity in the Anthropocene. Ecological opportunity requires temporal overlap among four conditions: niche availability, niche discordance, selection consistency, and biodiversity change potential. Green indicates conditions that persist long enough to support biodiversity change; red indicates transient conditions; gray indicates conditions that have already disappeared before they can be acted upon. Biodiversity change potential refers to the capacity of populations or communities to convert ecological opportunity into realized biodiversity change through standing genetic variation, plasticity, dispersal, demographic recovery, species sorting, and microbiome-mediated functional shifts. Under relatively stable Holocene conditions, these components were more likely to persist over ecological and evolutionary timescales. Under Anthropocene conditions, long-term directional trends are increasingly superimposed on short-term extremes, habitat fragmentation, interaction rewiring, and recurrent disturbance, shortening the temporal overlap among these components. The figure is conceptual and does not imply uniform global dynamics; opportunity compression is expected to vary geographically and to be strongest in systems where anthropogenic change is rapid, recurrent, and spatially pervasive.

For ecological opportunity to generate diversification, four interdependent conditions must be satisfied—and, critically, must persist long enough for evolution to act (Fig. 1). These conditions unfold across two temporal domains: Ecological time, during which populations may rapidly shift their realized niches through plasticity, including behavioral plasticity (West-Eberhard, 2008), or niche partitioning in response to new resources or reduced competition. Evolutionary time, during which natural selection can gradually alter the fundamental niche by modifying physiological, morphological, or behavioral traits, enabling access to new ecological space. If any condition fails, or if ecological opportunity is too transient, the pathway to diversification closes.

### Condition 1: niche availability (ecological)

Ecological opportunity begins with access to unoccupied or underused niche space. On ecological timescales, this can trigger rapid shifts in realized niche through dispersal and/or plasticity. Yet in the Anthropocene, niche space may open and close within days or years—faster than most organisms can respond. These absolute timescales hold very different consequences for different organisms: a transient window may span many generations for microbes or short-lived invertebrates, but less than one or only a few generations for long-lived organisms, such as vertebrates or plants. Even when opportunity arises, it may be immediately seized upon by rapid processes such as microbiome reorganization (Ziegler et al, 2019; Berg and Cernava, 2022; Voolstra and Ziegler, 2020), colonization by generalist species (Carvajal-Quintero et al, 2026; Pagani-Núñez et al, 2019; Sriswasdi et al, 2017), or short-lived resource pulses (Holt, 2008), leaving little temporal or ecological room for diversification.

### Condition 2: niche discordance and diversifying selection (ecological to evolutionary)

When unoccupied niches persist, a mismatch arises between environmental conditions and population traits—a state of niche discordance that drives diversifying selection and fuels adaptive change. Under Anthropocene dynamics, however, discordance is fleeting. Environmental variability (Calvin et al, 2023; Laurance et al, 2014; Coumou and Rahmstorf, 2012; Haddad et al, 2015)—heatwaves, flooding, fragmentation—can shift optimal traits across days to years, often faster than selection can act because adaptive change requires selection across reproductive cycles. Paradoxically, the same mechanisms that generate discordance, such as biological invasions (Wiens et al, 2019) and microbial turnover (Ziegler et al, 2019; Röthig et al, 2016), can also buffer populations against selective stress. By enhancing functional redundancy, plasticity, or resource availability, these processes may increase short-term persistence, potentially preserving populations long enough for selection to act. However, such buffering reduces fitness differences among genotypes, thereby weakening selection and dampening the very adaptive divergence these processes could otherwise promote (Ghalambor et al, 2007; Fox et al, 2019).

### Condition 3: selection consistency (evolutionary)

Diversification requires that selection maintains consistent form and direction across time and space. In relatively stable settings, such directional and temporal consistency can emerge. Under accelerating environmental change, however, long-term directional trends such as warming are increasingly superimposed on short-term extremes, spatial heterogeneity, and temporal fluctuations, repeatedly altering the selective landscape itself. As a result, weather extremes, pollutants, and land-use transitions can repeatedly reset trait optima and change which phenotypes are favored. When the selective optimum moves faster than populations can track, adaptive trajectories fracture or stall (Salinas et al, 2019), leaving populations locked in maladaptation, persistence without diversification, or collapse.

### Condition 4: biodiversity change potential

Even sustained opportunity and stable selection regimes cannot yield diversification without sufficient genetic and phenotypic variation, i.e., response capacity. We refer to this capacity as Biodiversity Change Potential (BCP): the availability of genetic, phenotypic, demographic, and ecological variation that allows populations and communities to convert niche availability and niche discordance into realized change. At the evolutionary level, BCP depends on standing genetic variation, mutation, recombination, gene flow, and admixture. At the ecological level, it is shaped by dispersal, demography, species sorting, phenotypic plasticity, and microbiome-mediated functional shifts. Processes that redistribute or amplify variation—gene flow, admixture—can transiently replenish adaptive potential, but without selection consistency, this input dissipates before diversification takes hold. In the Anthropocene, BCP is rapidly eroding as habitat fragmentation, demographic decline, and genetic drift reduce the raw material for selection. Strong directional pressures—from global warming to agricultural intensification—further narrow trait variation within and among species (McLean et al, 2019, 2022; Politano et al, 2026). Many systems show evidence of shifts towards more generalist strategies (MacNeil et al, 2025; Rocha et al, 2017; Peters et al, 2022). We predict that this convergence reduces the ecological and genetic heterogeneity needed for diversification to occur.

## The Anthropocene: compressing time and destabilizing opportunity

Although the start of the Anthropocene is debated, the profound and unprecedented impact humans have had on the environment is indisputable (Fig. 1) (Steffen et al, 2011; Subramanian, 2019). In Hutchinsonian terms, the Anthropocene destabilizes both realized niche space—the immediate ecological domain populations occupy—and fundamental niche space—their long-term evolutionary potential (Kassen et al, 2004; Rainey and Travisano, 1998). Previously, niche availability was often sufficiently stable relative to organismal generation times, dispersal, and demographic recovery for ecological sorting and adaptive divergence to unfold. Under Anthropocene conditions, however, niche availability has become highly dynamic and transient. Habitat loss, climate extremes, and biological invasions open niche space rapidly, sometimes within days or years, yet close it just as quickly (Pack et al, 2022; Jacobs et al, 2019). Diversification has become a race against time: opportunities emerge but rarely persist long enough for an adaptive response to manifest. Critically, the limiting factor is not time alone—often claimed to be a major deterrent to an adaptive response—but environmental consistency–sustained regimes that allow trajectories to consolidate.

Niche discordance remains the engine of evolutionary change, but in the Anthropocene, such mismatches are remarkably short-lived. Rapid abiotic shifts, whether heatwaves or salinity fluctuations, can alter optimal trait values faster than selection can act (Parmesan and Yohe, 2003; Hoffmann and Sgrò, 2011). Invasive species further disrupt interaction networks (Sorte et al, 2016), forcing resident species into evolutionary whiplash. These mismatches generate ecological opportunities that tend to collapse before divergence. Ironically, the same mechanisms that create discordance can also buffer it. Thus, although invasions, microbial turnover, and community reassembly may lead to niche mismatches, compensatory responses through functional redundancy, altered resource access, or plasticity can reduce fitness differences among genotypes and weaken the selective gradients required for adaptive divergence.

For diversification to proceed, selection must persist consistently across space and time. Under Anthropocene instability, this requirement is rarely met. Climate extremes, pollution pulses, and land-use shifts reshape selective pressures so frequently that no trait remains optimal for long (Findell et al, 2017; Smith et al, 2016). Adaptive trajectories shatter or stagnate, producing persistence without diversification. Rare stable niches—for example, those maintained by urban or managed environments (Johnson and Munshi-South, 2017; Donihue and Lambert, 2015; Santangelo et al, 2018)—may provide sufficient stability for evolutionary adaptation, but most ecological contexts have become too ephemeral for evolution to take hold.

Even when opportunity and selection align, we hypothesize that the potential for diversification is waning. Fragmentation (Haddad et al, 2015; Laurance et al, 2014), population decline (Dirzo et al, 2014; Hallmann et al, 2017; Bowler et al, 2019; Morgan et al, 2020), and genetic drift shrink the raw material on which selection can act. Evolutionary rescue can prevent extinction when adaptive variation is available and environmental change proceeds slowly enough for beneficial variants to spread (Lindsey et al, 2013; Bell and Gonzalez, 2009). However, under rapid or recurrent Anthropocene change, the same processes that erode population size, connectivity, and allelic diversity also reduce the probability that rescue can occur. The loss of allelic diversity constrains adaptive pathways, while strong directional pressures drive trait convergence toward generalist strategies. This functional homogenization constricts the evolutionary landscape, reducing the scope for ecological divergence and diversification.

Human influence also reverberates through ecological interactions (Brodie et al, 2014; Palmer et al, 2008; Toby Kiers et al, 2010; Pringle et al, 2019). Mutualistic networks—pollination, symbiosis, microbiome assembly—are unraveling, erasing both present functionality and future potential (Bell et al, 2019; Hegland et al, 2009), or shifting towards consumer-resource interactions (Becks et al, 2025). Extinction debts are rising, with species persisting temporarily despite conditions that no longer support long-term survival. At the same time, facilitation debts may also accumulate, i.e., unrealized or delayed positive interactions that, if stabilized, could support recovery or rekindle diversification (Henriques and Osmond, 2020). Rewired communities occasionally produce novel mutualisms, including between native and non-native species, transiently expanding realized niche space (Valido et al, 2019; Iwasaki and Hogendoorn, 2022), yet these arrangements are often context-dependent. Competitive interactions can stabilize coexistence by limiting positive feedbacks and weakening effective interaction strengths, particularly in microbial communities (Coyte et al, 2015). However, when rewiring increases interaction turnover, asymmetric competitive dominance, predation, parasitism, or shifts in fitness rankings, it can accelerate ecological turnover and further destabilize selection (Bøhn et al, 2008).

## From opportunity to persistence: bridging timescales

Microbiome-mediated plasticity adds a distinct fast-response mechanism, but it is only one of several processes that can buffer populations under rapid environmental change. Microbial communities can reorganize on short ecological timescales, often within days, potentially buffering hosts against environmental stress (Ziegler et al, 2019; Ziegler et al, 2017; Gillingham et al, 2025), but they may also deteriorate host condition (Hanski et al, 2012). Yet this plasticity is often reversible and fragile under repeated disturbance. Even when microbiome-associated states are passed to offspring, their persistence depends on transmission fidelity, vertical inheritance, host filtering, and environmental reinforcement (Baldassarre et al, 2022; Murphy et al, 2023; Hanski et al, 2012). It therefore provides short-term persistence more readily than long-term adaptation. By offering temporary physiological relief, plasticity can even reduce the strength of selection by reducing fitness differences between genotypes, creating a feedback that short-circuits evolutionary change (Fox et al, 2019).

As ecological dynamics, including microbial dynamics, interact, niche discordance becomes both amplified and transient. The arrival of new competitors, predators, or mutualists can displace established equilibria (Paine, 1966), generating new axes of mismatch (Elliott et al, 2021). Likewise, changes in microbiome composition can shift host phenotypes (Moeller et al, 2020), either intensifying or buffering discordance. These fluctuations can spark diversifying selection—but only if environments stabilize long enough to allow heritable adaptation to accumulate.

Selection consistency remains the most vulnerable condition under rapid change because shifting environmental pressures repeatedly alter which traits are favored. Constant ecological turnover reshapes species interactions and environmental conditions, causing the optimal phenotype to shift rapidly. As a result, evolution faces a moving target. While microbiome-mediated  plasticity can momentarily stabilize niche occupation, we posit that it can also blunt cumulative selection pressures, giving the false appearance of adaptation where none endures (Kolodny and Schulenburg, 2020).

Diversification potential hinges on the generation and maintenance of variation across scales. While mutation and recombination remain essential, ecological processes—such as migration (i.e., dispersal among populations, including emigration from a source population and successful establishment at a novel site, both of which can contribute to gene flow), demographic shifts, and species turnover—rapidly reshape the pool of available diversity (Uecker and Hermisson, 2016; Hermann and Becks, 2022; Ceballos et al, 2017). Immigrating species may occupy open niches before residents can diversify locally, favoring generalists that thrive in flux while specialists are pruned away.

Mutualistic networks—from plant–pollinator systems to host–microbe symbioses—can help maintain diversity when interaction partners remain available, functionally redundant, and temporally synchronized. Under these conditions, mutualisms can buffer small populations and preserve genetic variation. However, this buffering capacity also creates dependence: when key partners are lost, interaction timing shifts, or network structure breaks down, formerly stabilizing interactions can become sources of vulnerability. Such breakdowns can trigger extinction debts (Kuussaari et al, 2009), while network reassembly may take decades, falling out of sync with the pace of environmental change. The resulting temporal lag can erase lineages before new associations arise. Fast microbial and ecological responses thus determine whether opportunity persists long enough for evolution to act upon. By buffering stress, maintaining population size, or stabilizing host function, these rapid responses can extend the demographic window in which selection can operate. At the same time, if microbial turnover, community rewiring, or plasticity rapidly shift trait–environment relationships, they can also shorten or redirect this window before genetic adaptation accumulates. Opportunity therefore depends not only on whether new niches arise but also on whether fast ecological responses stabilize them long enough to become evolutionary substrates.

## Rebuilding the engine of biodiversity

**Figure. 2:**
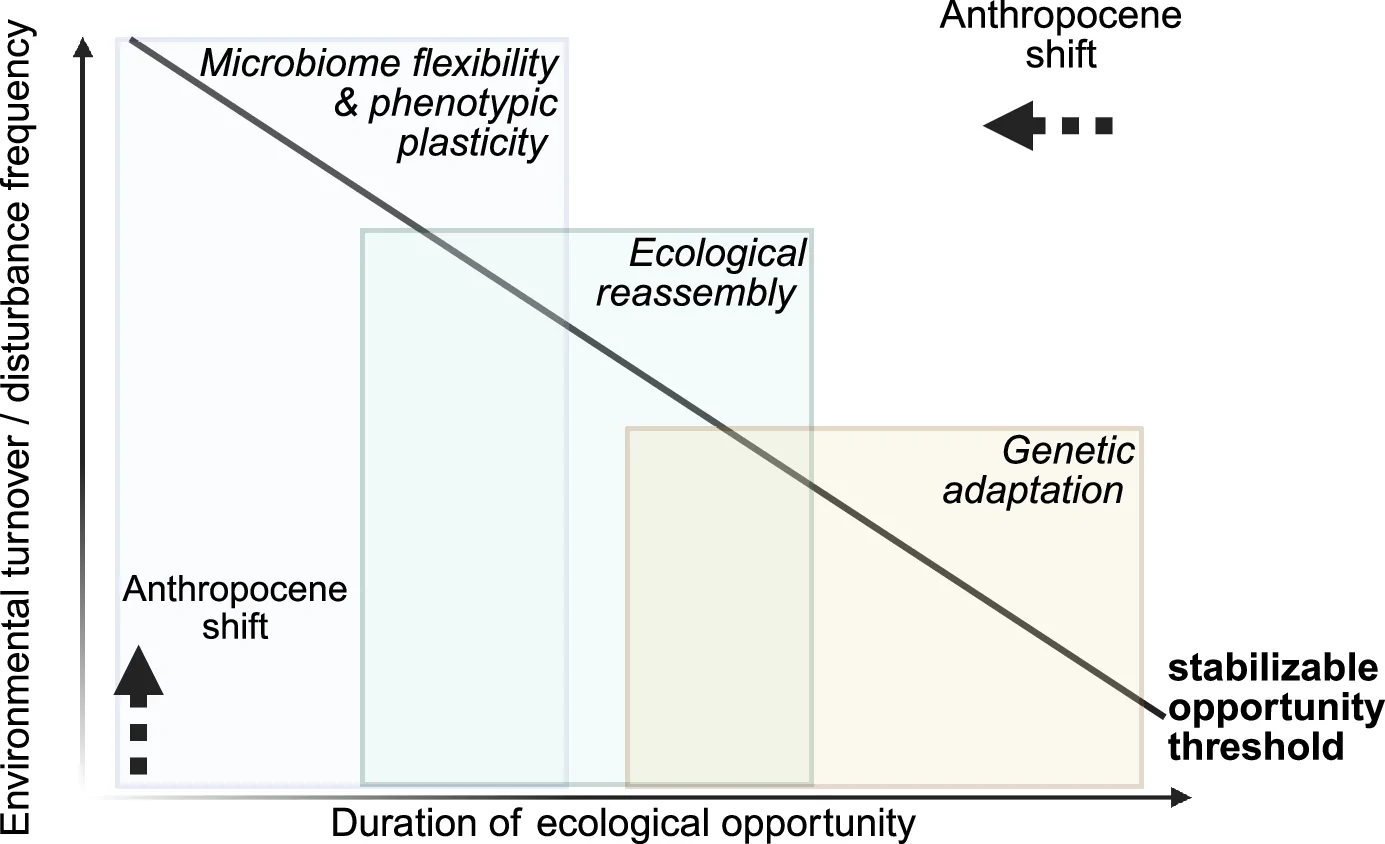
Stabilizing ecological opportunity in a rapidly changing world. Whether ecological opportunity translates into persistence or evolutionary change depends on its temporal stability relative to the pace of environmental turnover. The diagonal boundary denotes the stabilizable opportunity threshold: above it, opportunities are too transient for adaptive processes to act, resulting in short-lived ecological novelty without lasting evolutionary consequences. Below the boundary, opportunities persist long enough to enable persistence, niche expansion, and diversification. Different biological response mechanisms operate across distinct temporal domains: microbiome flexibility underlies microbiome-mediated plasticity, while behavioral and physiological responses constitute phenotypic plasticity, both of which can respond rapidly to acute environmental shifts; ecological reassembly—here defined as non-genetic shifts in dispersal, community composition, species interactions, trophic structure, or habitat use—can support intermediate responses; and genetic adaptation and diversification require the longest periods of environmental consistency. In the Anthropocene, accelerating environmental change shifts ecosystems toward conditions of higher instability and shorter opportunity duration, pushing systems above the stabilizable opportunity threshold and constraining the capacity for evolutionary responses. Quantifying these temporal thresholds is therefore essential for predicting when ecological opportunity can translate into biodiversity persistence or diversification.

Rebuilding biodiversity in the Anthropocene requires moving beyond classical evolutionary assumptions toward a timescale-integrated framework (Fig. 2). Unlike ecological succession or post-disturbance assembly, the Anthropocene offers no stable baseline—niche availability remains transient, selection is inconsistent, and genetic variation is declining under temporal compression.

Bridging evolutionary biology and ecology with microbiome science as a key integrative dimension reveals pathways forward. Ecological dynamics—dispersal, demographic change, community turnover—restructure niche availability rapidly, while microbiome-mediated plasticity enables host phenotypic shifts that buffer acute stress. Yet this plasticity remains reversible and disturbance-fragile, often blunting selection and delaying, rather than driving, long-term adaptation. Restoring adaptive capacity requires more than buffering instability; it hinges on reconnecting evolutionary reservoirs across scales. Rebuilding biodiversity change potential requires maintaining the capacity for genetic and microbial exchange—through population connectivity, hybridization, and microbial symbioses—so that selection acts on a sufficiently varied substrate when opportunity stabilizes.

Biodiversity science must therefore confront a defining question: when does ecological opportunity persist long enough to matter? The answer demands a predictive framework integrating realized–fundamental niche dynamics, ecological–evolutionary timescales, and microbiome flexibility across temporal domains (Box 1). This requires shifting conservation from species preservation to evolutionary scaffolding: maintaining genetic reservoirs through connectivity and supplementation, ensuring selection continuity via stabilized refugia and corridors, and leveraging microbiome resilience selectively to extend—but not over-buffer—opportunity windows. Strategic interventions—habitat networks, assisted migration, microbial inoculation, and refugia management—show promise but succeed only when paired with environmental stability. By stabilizing temporal alignment and selection consistency, we restore not just biodiversity, but life’s capacity to diversify under flux. Plasticity is valuable because it keeps populations alive, but the time it buys becomes evolutionarily meaningful only if opportunity persists long enough to be converted into heritable change. The challenge is therefore not to choose between plasticity and evolution, but to recognize their different temporal roles: plasticity can preserve populations through transient disturbance, whereas enduring biodiversity requires ecological opportunity to be stabilized across generations. In the Anthropocene, safeguarding biodiversity means maintaining not only species and interactions but also the temporal conditions under which adaptation and diversification remain possible. Plasticity buys time, but evolution forges enduring biodiversity.

Box 1 Research priorities: empirical thresholds to stabilizable ecological opportunity Understanding whether ecological opportunity can translate into diversification requires quantifying the temporal conditions under which adaptive processes can operate. We highlight four empirical thresholds that biodiversity science must establish.

1. **Opportunity realization thresholds**
  Quantify the minimum duration that a novel ecological opportunity must persist for selection to act. This requires linking environmental variance, disturbance frequency, and niche availability to the timescales of population persistence and demographic recovery.
2. **Ecological–evolutionary response thresholds**
  Determine the temporal window required for adaptive responses across mechanisms, including phenotypic plasticity, microbiome-mediated plasticity, and genetic adaptation. Establish comparative estimates of response rates across taxa and environments.
3. **Realized–fundamental niche divergence thresholds**
  Identify when environmental variability repeatedly collapses the realized niche before populations can expand toward the fundamental niche, i.e., when environmental change happens too quickly for species to expand their realized niche. This requires empirical tests linking niche breadth, environmental volatility, and persistence.
4. **Microbiome-mediated buffering thresholds**
  Quantify the extent to which microbiome flexibility enables microbiome-mediated plasticity that can stabilize host performance under fluctuating conditions. Critical questions include how rapidly microbial communities reorganize, how long functional buffering persists, when these effects fail under sustained stress, and when microbiome-mediated plasticity reduces selective pressure on the host, slowing or even preventing genetic adaptation.
5. **Opportunity–instability tipping points**
  Identify tipping points where environmental turnover exceeds the combined buffering capacity of phenotypic plasticity, microbiome-mediated plasticity, and evolutionary adaptation. Crossing this boundary transforms opportunity from a driver of diversification into a transient ecological anomaly.

## Supplementary information

Peer Review File
