# Supplementary material for: Timescale mismatch: redefining ecological opportunity for evolution in the Anthropocene
Source: EMBO J. 2026 Jul 31;45(18):6359–66. doi: 10.1038/s44318-026-00880-3 (PMC13578450; doi:10.1038/s44318-026-00880-3)
Supplement: Supplementary file 1 [file 44318_2026_880_MOESM1_ESM.pdf]

THE EMBO JOURNAL

Timescale mismatch: redefining ecological opportunity for evolution in the Anthropocene

Christian Voolstra, Ruben Hermann, Herwig Stibor, Alexander Keller, and Lutz Becks

Corresponding author(s): Christian Voolstra (christian.voolstra@uni-konstanz.de), Lutz Becks (lutz.becks@uni-konstanz.de)

Review Timeline:

Submission Date:	30th Apr 26
Editorial Decision:	19th Jun 26
Revision Received:	13th Jul 26
Accepted:	14th Jul 26

Editor: Ioannis Papaioannou

Transaction Report:

(Note: With the exception of the correction of typographical or spelling errors that could be a source of ambiguity, letters and reports are not edited. Depending on transfer agreements, referee reports obtained elsewhere may or may not be included in this compilation. Referee reports are anonymous unless the Referee chooses to sign their reports.)

1st Editorial Decision 19th Jun 2026

Dear Christian,

Thanks again for submitting your manuscript (EMBOJ-2026-124512P) to The EMBO Journal for our consideration, and for your patience during peer review. Please excuse the rather protracted process on this occasion - the reasons for the delay were some initial difficulties in securing experienced referees with suitable expertise, and then extensive conference travel obligations in our team and a related backlog in our office in the last few weeks. Many thanks for your patience and understanding.

Your manuscript has been seen by two experts in the field, and we have now received their comments, which are appended below. As you will see, they are both largely supportive, with their comments pointing to the conclusion that this is a novel and relevant piece that should be of interest to the community. Along with their reports, both referees indicated in the separate evaluation sheets we always ask our referees to return along with their comments that the quality, novelty/timeliness, and level of general interest are all "high", the length suitable, and their recommendation is to consider this piece further for publication in The EMBO Journal pending minor textual revisions. In light of this input and our editorial discussions, I am happy to inform you that your piece has been accepted in principle for publication in The EMBO Journal.

As you will see in the detailed comments of the referees (below), they also provide lists of specific suggestions for further stregthening of the manuscript, including a few minor corrections, additions, clarifications, and improvement of the presentation, structuring, and flow of the text. We kindly request that you consider these suggestions carefully and revise your manuscript accordingly. We would specifically like to emphasize that all points with regard to better clarity and accessibility of the text to the non-specialists should be taken on board, as The EMBO Journal has a very broad readership of molecular biologists working across all areas of molecular life sciences. Please also submit along with your revised manuscript a point-by-point response to the referees' comments and suggestions.

We have also discussed your manuscript and the referees' reports within our editorial team, and we think that the best-fitting article type for it is that of a Commentary. In order to avoid any further delays in the handling of the manuscript and its processing for publication, we would be grateful if you could please address the following editorial requests for changes in the final version of the manuscript:

- Please upload the main manuscript file in .docx format, without Figures (these should be uploaded separately, as individual high-quality Figure files) and track changes.

- While Figures should be removed from the manuscript file and uploaded separately, their legends should remain in the manuscript, below the References list.

- Please make sure that bold text is used for the Abstract in the manuscript file.

- Please change heading "Funding" to "Acknowledgements" in the manuscript.

- The funding information that is provided in the Acknowledgements section of the manuscript should also be entered in the online manuscript handling system.

- Please rename heading "Competing interests" to "Disclosure and competing interests statement" in the manuscript.

- Please include in your "Disclosure and competing interests statement" section the statement: "Christian Voolstra is a member of the Advisory Editorial Board of EMBO Press. This has no bearing on the editorial consideration of this article for publication".

- The Author Contributions statement should be removed from the manuscript file. Instead, we use CRediT to specify the contributions of each author in the journal submission system. Please feel free to use the free text box to provide more detailed descriptions during submission. See also our guide to authors for more information: https://link.springer.com/partners/embo-press/editorial-policies#Authorship.

- The reference format must be updated to the style used by EMBO Press (i.e., alphabetical list, with the names of the first 10 co-authors listed for each citation, followed by "et al."). For more information, please refer to our Author Guidelines: https://link.springer.com/journal/44318/submission-guidelines#cms-Reference-guidelines.

- The order of the manuscript sections should be updated as follows: Title page - Abstract - Introduction - Acknowledgements - Disclosure and Competing Interests Statement - References - Figure Legends - Tables (if there are any).

- Please also note that as part of the EMBO Press transparent editorial process, The EMBO Journal publishes online a Peer Review File along with each accepted manuscript. This File will be published in conjunction with your Commentary and will include the referees' reports, your point-by-point responses and all pertinent correspondence relating to the manuscript. Please let us know in case you want to remove any data from your point-by-point responses before they are published as part of the

Peer Review File. Retaining unpublished data in the Peer Review File means that these count as published and that the Peer Review File would need to be referenced in future publications. Please let the editorial office know in case you want to remove any data from this file (contact@embojournal.org).

We look forward to seeing a final version of your manuscript as soon as possible. Please let us know if you have any questions and use this link to submit your revision: https://emboj.msubmit.net/cgi-bin/main.plex.

Best wishes,

Ioannis

Ioannis Papaioannou, PhD
Editor, The EMBO Journal
i.papaioannou@embojournal.org

Referee #1:

The manuscript by Voolstra et al. offers a timely and compelling synthesis of how the accelerated pace of environmental change in the Anthropocene reshapes the concept of ecological opportunity, emphasizing the critical role of timescale mismatches.
By clearly articulating four interdependent conditions that must persist for diversification-niche availability, niche discordance, selection consistency, and diversification potential-the authors provide a robust, testable framework for evaluating when fleeting opportunities can become lasting evolutionary drivers. The integration of microbiome-mediated plasticity as a rapid but reversible buffer against environmental changes adds an innovative dimension to classic ecological-evolutionary theory, highlighting both its strengths and its limits in the face of sustained disturbance.
Figures 1 and 2 effectively visualize the shift from a stable Holocene paradigm to a highly dynamic Anthropocene context, illustrating how opportunity duration relative to environmental turnover determines the likelihood of evolutionary persistence versus mere ecological novelty. The authors' "timescale-integrated framework" is well-grounded in current literature, linking empirical observations of rapid niche openings after disturbances with the increasingly transient nature of these windows in modern ecosystem.
Box 1 concrete research priorities, such as quantifying opportunity realization thresholds and microbiome-mediated buffering limits and offers clear guidance for future empirical work, making the manuscript highly actionable for the research community. The discussion thoughtfully points out that conservation strategies must shift from species preservation to "evolutionary scaffolding," emphasizing connectivity, refugia, and judicious use of microbial inoculation to stabilize selection regimes-an insight that bridges theory and practice. Overall, the paper presents a rigorous, interdisciplinary perspective that advances our understanding of biodiversity dynamics under rapid anthropogenic change and will stimulate fruitful research initiatives.

Specific points:

Line 59: Please consider and discuss vertical transmission of microbiomes as a potential mechanism to pass on microbiome mediated plasticity to the next generation, resulting in transgenerational effects (see e.g. https://doi.org/10.1038/s41467-022-31350-z)

Line 74: Please clarify which definition for plasticity you refer to: e.g. West-Eberhard 2008 defines behavior as part of plasticity (https://www.sciencedirect.com/topics/earth-and-planetary-sciences/phenotypic-plasticity)

Line 78: What do you mean by opportunity collapses? I would not argue with collapses, but rather with a continuous change of conditions, which might be faster than adaptation can take place.

Figure 1: I suggest to include the labeling of conditions also on the right side of the circle.

Line 99-102: However, one could also argue that plasticity generates time for selection by buffering the effects of stressful situations rather than eliminating them (which would otherwise lead to the collapse of populations).

Line 108-109: But aren't today's changes in conditions nevertheless moving in a consistent direction? However, the pace of change has accelerated dramatically, it is getting warmer, saltier, the pH level is dropping, etc.

Line 142: You're arguing with examples that relate to extreme local situations (which are indeed instable), but in my opinion, global trends are still moving in a consistent direction, just at a faster pace.

Line 158: not clear for me what you mean by "extinction debts" and "facilitation debts".

Line 162: However, several studies e.g in the human microbiome showed that high diversity of species is likely to coexist stably when the system is dominated by competitive, rather than cooperative, interactions (https://www.science.org/doi/10.1126/science.aad2602).

Line 170: Why non-heritable? Bacteria can be vertically transmitted to the next generation.

Line 172: However, plasticity could also mitigate stress conditions, allowing selection to act, before populations collapse.

Line 197: Exactly! I would suggest to elaborate this point.

Line 209: As pointed out before, please consider vertical bacterial transmission in your argumentation.

Line 227: This point should be considered more prominently in the manuscript.

Referee #2:

This is an interesting, thought-provoking contribution about ecological and evolutionary time compressing and ecological opportunity destabilizing in the Anthropocene. The authors deliver an important take-home message on a time-scale integrated framework for dealing with eco-evolutionary change and biodiversity loss. Overall, I support their warnings and the issues addressed. However, I believe at some places their manuscript needs to be better underpinned with references or should be more elaborate so that the reader can clearly follow all reasonings and arguments. At some places, I would recommend to modify the tone of the manuscript into predictions and working hypotheses rather than bold statements as it stands in this version of the manuscript. As far as I am concerned, slightly more cautious wording does not detract from the importance of this piece but reflects careful scholarly writing also in a forum-lie contribution.

Below I list a number of specific comments, mostly in line with my general comment on style and underpinning rather than on the real content of this manuscript, which is interesting and highly relevant.

1) L. 1: Title - I would consider referring explicitly to evolution in the title as this is a key element and is currently not really reflected in the phrasing used. May be relevant to attract a broader readership.
2) L. 42: be explicit here that you refer to generations of the organisms under consideration.
3) L. 62: Add reference(s) here.
4) L. 82: the notion of facilitating ecological opportunity and how it is expressed on this figure should be elaborated in a clearer way, I would argue. Particularly the notion of 'biodiversity change potential' remains too vague to the reader. Moreover, is this figure and its conclusion valid worldwide, or biased to the most anthropogenic areas of the world including (NW)-Europe? This needs some treatment as well, I believe.
5) L. 89: 'days or years' need some further underpinning (clear examples/references) and of course there are quite a lot of organisms with short life cycles relative to our time perspective, think about several insects and other invertebrates. So, here some more elaboration to make the point more convincing would be useful.
6) L. 99: '...faster than selection can act.' Add some words why this is the case.
7) L. 102-104: Please underpin this with references.
8) L. 109: Be precise what is meant by 'no consistent axis of selection'? Is this always by definition directional selection? I don't think so.
9) L. 121: Add reference or phrase it as a prediction/working hypothesis rather than a certitude per se.
10) L. 127: How would you define 'relative sable' here? The reader needs to have some assistance here.
11) L. 137: I am confused about the use of reference 46 as it refers to beneficial effects of invasive/exotic species, if I understood correctly.
12) L. 140: Could use a bit more elaboration to make your point.
13) L. 149-150: Add reference or phrase it as a prediction/working hypothesis rather than a certitude per se.
14) L. 151: I am not convinced that referring to evolutionary rescue only between brackets here is an optimal choice. Consider incorporating it more explicitly in the main text.
15) L. 158: Extinction debt likely is known by the majority of the readership, but I doubt whether it is also the case for 'facilitation debt'. Hence, I suggest adding a definition.
16) L. 161-162: "Yet these arrangements are often unstable;...". Add reference or phrase it as a prediction/working hypothesis rather than a certitude per se.
17) L. 168-169: That issue needs a more carefully discussion as there is evidence since a decade that microbial diversity may be

correlated with other biodiversity components (e.g. Hanski et al 2012. PNAS).
18) L. 169-170: Add reference or phrase it as a prediction/working hypothesis rather than a certitude per se.
19) L. 173: Add reference(s).
20) L. 174: I find it somewhat strange to make use of the different labels 'ecological' and 'microbial' as microbes also are involved in ecological dynamics.
21) L. 180: Add concisely why that is the case.
22) L. 184: Add reference(s)
23) L. 192-194: Not clear to the reader as you indicate the buffering capacity on the one hand, but the fragile nature on the other. Needs elaboration to clearly inform the reader about this kind of paradox.
24) L. 206: 'migration'. In the scientific literature there is a confusing use of the term as you mean 'dispersal' here and not 'migration' as a behavioural ecologist would interpret it as a seasonal movement without gene flow consequences. I know as you discuss further also 'assistant migration' (L.224) (which again is about dispersal), I would suggest at least the first time you refer to migration to clarify that you refer by migration to dispersal (with emigration as key component here).
25) L. 229: Figure 2. I have some doubts about the use of 'ecological adjustment'; at least it needs a clear definition.

Referee #1:

The manuscript by Voolstra et al. offers a timely and compelling synthesis of how the accelerated pace of environmental change in the Anthropocene reshapes the concept of ecological opportunity, emphasizing the critical role of timescale mismatches.

By clearly articulating four interdependent conditions that must persist for diversification-niche availability, niche discordance, selection consistency, and diversification potential-the authors provide a robust, testable framework for evaluating when fleeting opportunities can become lasting evolutionary drivers. The integration of microbiome-mediated plasticity as a rapid but reversible buffer against environmental changes adds an innovative dimension to classic ecological-evolutionary theory, highlighting both its strengths and its limits in the face of sustained disturbance.

Figures 1 and 2 effectively visualize the shift from a stable Holocene paradigm to a highly dynamic Anthropocene context, illustrating how opportunity duration relative to environmental turnover determines the likelihood of evolutionary persistence versus mere ecological novelty. The authors' "timescale-integrated framework" is well-grounded in current literature, linking empirical observations of rapid niche openings after disturbances with the increasingly transient nature of these windows in modern ecosystem.

Box 1 concrete research priorities, such as quantifying opportunity realization thresholds and microbiome-mediated buffering limits and offers clear guidance for future empirical work, making the manuscript highly actionable for the research community.

The discussion thoughtfully points out that conservation strategies must shift from species preservation to "evolutionary scaffolding," emphasizing connectivity, refugia, and judicious use of microbial inoculation to stabilize selection regimes-an insight that bridges theory and practice. Overall, the paper presents a rigorous, interdisciplinary perspective that advances our understanding of biodiversity dynamics under rapid anthropogenic change and will stimulate fruitful research initiatives.

Response: We thank the reviewer for this highly positive assessment of our manuscript and for recognizing the value of the timescale-integrated framework.

Specific points:

Line 59: Please consider and discuss vertical transmission of microbiomes as a potential mechanism to pass on microbiome mediated plasticity to the next generation, resulting in transgenerational effects (see e.g. https://doi.org/10.1038/s41467-022-31350-z)

Response: Thank you. This is a very good point and the study, and its implications have been added to the revised manuscript.

Revised text reads: "Still, this adaptive flexibility is often temporary and easily disrupted by ongoing disturbance. However, recent work suggests that microbiome-mediated flexibility can, in certain cases, be transmitted to subsequent generations, as offspring may harbor specific members of acclimated microbiota (Baldassarre et al, 2022). Even when microbiome-associated states are inherited, their persistence depends on transmission fidelity, host filtering, and environmental reinforcement. Thus, rather than substituting for evolution, it dampens selective gradients, buffering stress effectively and thereby short-circuiting genetic adaptation in many systems (Ghalambor et al, 2007; Fox et al, 2019).

Line 74: Please clarify which definition for plasticity you refer to: e.g. West-Eberhard 2008 defines behavior as part of plasticity (https://www.sciencedirect.com/topics/earth-and-planetary-sciences/phenotypic-plasticity)

Response: Thank you. We agree that behavior is considered part of phenotypic plasticity, following West-Eberhard (2008). Our intention was to make this component explicit, as readers may more readily associate plasticity with physiological changes.

Revised text reads: "Ecological time, during which populations may rapidly shift their realized niches through plasticity, including behavioral plasticity (West-Eberhard, 2008), or niche partitioning in response to new resources or reduced competition."

Line 78: What do you mean by opportunity collapses? I would not argue with collapses, but rather with a continuous change of conditions, which might be faster than adaptation can take place.

Response: Correct, we meant to indicate the absence of *stable* (ecological) opportunity.

Revised text reads: "If any condition fails, or if ecological opportunity is too transient, the pathway to diversification closes."

Figure 1: I suggest to include the labeling of conditions also on the right side of the circle.

Response: Good point. This has been implemented in the revised figure.

Line 99-102: However, one could also argue that plasticity generates time for selection by buffering the effects of stressful situations rather than eliminating them (which would otherwise lead to the collapse of populations).

Response: Thank you for raising this point. As it goes to one of the core arguments in this manuscript, it's important to get this right. We agree that plasticity can buffer stressful conditions and thereby increase *short-term population persistence*, which may in some cases preserve the demographic substrate on which selection can subsequently act. Our intended point was not that plasticity necessarily eliminates selection, but that buffering can *reduce the strength of selective gradients* when it diminishes fitness differences

among genotypes or phenotypes. We have revised the text to clarify this distinction between plasticity buying demographic time and plasticity dampening adaptive divergence.

Revised text reads: "By enhancing functional redundancy, plasticity, or resource availability, these processes may increase short-term persistence, potentially preserving populations long enough for selection to act. However, such buffering reduces fitness differences among genotypes, thereby weakening selection and dampening the very adaptive divergence these processes could otherwise promote."

Line 108-109: But aren't today's changes in conditions nevertheless moving in a consistent direction? However, the pace of change has accelerated dramatically, it is getting warmer, saltier, the pH level is dropping, etc.

Response: We agree that many anthropogenic drivers show a consistent directional trend, for example warming, acidification, or salinization. Our intended point was that directional environmental change does not necessarily translate into selection consistency. Selection must maintain a sufficiently stable form across time and space for diversification to proceed. Increasing frequency and intensity of extremes, spatial heterogeneity, and temporal fluctuations can repeatedly alter which traits are favored, even when the long-term environmental trend is directional. We have revised the text to clarify this distinction.

Revised text reads: "Diversification requires that selection maintain consistent form and direction across time and space. In relatively stable settings, such directional and temporal consistency can emerge. Under accelerating environmental change, however, long-term directional trends such as warming are increasingly superimposed on short-term extremes, spatial heterogeneity, and temporal fluctuations, repeatedly altering the selective landscape itself. As a result, weather extremes, pollutants, and land-use transitions can repeatedly reset trait optima and change which phenotypes are favored."

Line 142: You're arguing with examples that relate to extreme local situations (which are indeed instable), but in my opinion, global trends are still moving in a consistent direction, just at a faster pace.

Response: Thank you. As outlined above, although long-term trends are (at large) directional, they are superimposed on short-term extremes, spatial heterogeneity, and temporal fluctuations, which can repeatedly alter the selective landscape itself by shifting which traits are favored.

Line 158: not clear for me what you mean by "extinction debts" and "facilitation debts".

Response: The phrase "extinction debt" is established and means species are still present, but already committed to future extinction because habitat, population size, or interactions have deteriorated below what is needed for long-term persistence. Facilitation debt is our

analogous concept and refers to the notion that positive interactions that could support recovery or diversification are delayed, unrealized, or not yet stabilized. We agree the term is not familiar, and we have added definitions for both terms to the revised text.

Revised text reads: "Extinction debts are rising, with species persisting temporarily despite conditions that no longer support long-term survival. At the same time, facilitation debts may also accumulate, i.e. unrealized or delayed positive interactions that, if stabilized, could support recovery or rekindle diversification (Henriques & Osmond, 2020)."

Line 162: However, several studies e.g in the human microbiome showed that high diversity of species is likely to coexist stably when the system is dominated by competitive, rather than cooperative, interactions (https://www.science.org/doi/10.1126/science.aad2602).

Response: Thank you for this important clarification. We agree that competitive interactions are not inherently destabilizing and that, particularly in microbiomes, competition can stabilize coexistence by limiting positive feedbacks and weakening effective interaction strengths. Our intended point was not that antagonistic interactions universally destabilize communities, but that interaction rewiring under rapid environmental change can destabilize selection when it increases interaction turnover, asymmetric dominance, predation, parasitism, or context-dependent changes in which species or traits are favored. We have revised the text accordingly to avoid implying that competition per se is destabilizing.

Revised test reads: "Yet these arrangements are often context-dependent. Competitive interactions can stabilize coexistence by limiting positive feedbacks and weakening effective interaction strengths, particularly in microbial communities (Coyte et al, 2015). However, when rewiring increases interaction turnover, asymmetric competitive dominance, predation, parasitism, or context-dependent shifts in fitness rankings, it can accelerate ecological turnover and further destabilize selection (Bøhn et al, 2008).

Line 170: Why non-heritable? Bacteria can be vertically transmitted to the next generation.

Response: Thank you for this helpful clarification. It is great to have a microbiome expert reviewer to sharpen our manuscript, making it more salient. We agree that original phrase was too absolute. Our intention was to emphasize that microbiome-mediated plasticity is generally more reversible and condition-dependent than genetic inheritance. We have revised the text to avoid the categorical statement that microbiome-mediated plasticity is non-heritable.

Revised text reads: "Yet this flexibility is often reversible and fragile under repeated disturbance. Even when microbiome-associated states are passed to offspring, their persistence depends on transmission fidelity, the extent to which microbiota are vertically inherited, host filtering, and environmental reinforcement (Baldassarre et al, 2022; Murphy

et al, 2023; Hanski et al, 2012). It therefore provides short-term persistence more readily than long-term adaptation."

Line 172: However, plasticity could also mitigate stress conditions, allowing selection to act, before populations collapse.

Response: As alluded above (response to Line 99-102), our intended point was not that plasticity necessarily eliminates selection, but that buffering can reduce the *strength of* selective gradients when it diminishes fitness differences among genotypes. In the revised text we make this more explicit.

Revised text reads: "By offering temporary physiological relief, plasticity can even reduce the strength of selection by reducing fitness differences between genotypes, creating a feedback that short-circuits evolutionary change.

Line 197: Exactly! I would suggest to elaborate this point.

Response: Thank you. We have elaborated the sentence to make the argument explicit. Fast microbial and ecological responses can shape the duration of ecological opportunity in two opposing ways: they may extend the window for evolution by buffering stress and maintaining population persistence, but they may also shorten or redirect opportunity if they rapidly alter trait–environment relationships before genetic adaptation accumulates.

Added text reads: "Fast microbial and ecological responses thus determine whether opportunity persists long enough for evolution to act upon. By buffering stress, maintaining population size, or stabilizing host function, these rapid responses can extend the demographic window in which selection can operate. At the same time, if microbial turnover, community rewiring, or plasticity rapidly shifts trait–environment relationships, they can also shorten or redirect this window before genetic adaptation accumulates. Opportunity therefore depends not only on whether new niches arise, but on whether fast ecological responses stabilize them long enough to become evolutionary substrates."

Line 209: As pointed out before, please consider vertical bacterial transmission in your argumentation.

Response: Thank you. Since the sentence refers to all four mechanisms–i.e., migration, demographic change, community turnover, and microbiome flexibility–and to avoid implying that microbiome-mediated flexibility is categorically non-heritable, we have removed "non-heritable" from this sentence. Together with the additional clarification introduced earlier in the manuscript—that inherited microbiome-associated states depend on transmission fidelity, the extent of vertical inheritance, host filtering, and environmental reinforcement—we believe the revised text more accurately captures the conditional nature of microbiome transmission.

Revised text reads: "Yet this flexibility remains reversible and disturbance-fragile, often blunting selection and delaying rather than driving long-term adaptation."

Line 227: This point should be considered more prominently in the manuscript.

Response: Thank you. Motivated by your comments above, the distinction has been made more explicitly throughout the manuscript. The revised text clarifies that plasticity can buy demographic time by buffering stress and preventing population collapse, thereby preserving the substrate on which selection may act. At the same time, such buffering can weaken selective gradients when it reduces fitness differences among phenotypes. We have revised the end by explicitly reconnecting plasticity to the full timescale argument: plasticity is valuable because it keeps populations alive, but conservation should aim to stabilize the conditions under which that time can be converted into heritable change.

Added test reads: "By stabilizing temporal alignment and selection consistency, we restore not just biodiversity, but life's capacity to diversify under flux. Plasticity is valuable because it keeps populations alive, but this time only becomes evolutionarily meaningful if opportunity persists long enough to be converted into heritable change. The challenge is therefore not to choose between plasticity and evolution, but to recognize their different temporal roles: plasticity can preserve populations through transient disturbance, whereas enduring biodiversity requires ecological opportunity to be stabilized across generations. In the Anthropocene, safeguarding biodiversity means maintaining not only species and interactions but also the temporal conditions under which adaptation and diversification remain possible. Plasticity buys time but evolution forges enduring biodiversity. Plasticity buys time but evolution forges enduring biodiversity."

Referee #2:

This is an interesting, thought-provoking contribution about ecological and evolutionary time compressing and ecological opportunity destabilizing in the Anthropocene. The authors deliver an important take-home message on a time-scale integrated framework for dealing with eco-evolutionary change and biodiversity loss. Overall, I support their warnings and the issues addressed. However, I believe at some places their manuscript needs to be better underpinned with references or should be more elaborate so that the reader can clearly follow all reasonings and arguments. At some places, I would recommend to modify the tone of the manuscript into predictions and working hypotheses rather than bold statements as it stands in this version of the manuscript. As far as I am concerned, slightly more cautious wording does not detract from the importance of this piece but reflects careful scholarly writing also in a forum-like contribution.

Response: We thank Referee #2 for the positive and constructive assessment of our manuscript. We have revised the manuscript to better substantiate key arguments with additional references, to clarify reasoning where requested, and to adjust the tone where appropriate from categorical statements toward predictions, working hypotheses, and more cautious scholarly formulation. We agree that these changes improve the balance and accessibility of the manuscript while preserving its central message.

Below I list a number of specific comments, mostly in line with my general comment on style and underpinning rather than on the real content of this manuscript, which is interesting and highly relevant.

1) L. 1: Title - I would consider referring explicitly to evolution in the title as this is a key element and is currently not really reflected in the phrasing used. May be relevant to attract a broader readership.

Response: Thank you for this helpful suggestion. We agree that the evolutionary dimension should be more visible in the title. At the same time, we use "ecological opportunity" in its established sense as niche availability or niche discordance that can be seized through evolutionary adaptation and diversification. To preserve this distinction while making the evolutionary focus explicit, we have revised the title to: "Timescale mismatch: redefining ecological opportunity <u>for evolution</u> in the Anthropocene."

2) L. 42: be explicit here that you refer to generations of the organisms under consideration.

Response: Thank you for this clarification. We have revised the sentence to specify that "generations" refers to the generation times of the organisms under consideration.

Revised text reads: "Climate extremes unfold within days, landscapes fragment within years, and species <u>can</u> disappear within <u>only a few</u> generations <u>of the organisms concerned</u> (...)."

3) L. 62: Add reference(s) here.

Response: Thank you for requesting clarification. We have added references to the revised text to make clear that this argument builds on established phenotypic plasticity theory, where plastic responses can increase short-term persistence but may also reduce directional selection when they move phenotypes closer to local optima or reduce fitness differences among genotypes.

We have added the following references:

GHALAMBOR, C.K., McKAY, J.K., CARROLL, S.P. and REZNICK, D.N. (2007), Adaptive versus non-adaptive phenotypic plasticity and the potential for contemporary adaptation in new environments. Functional Ecology, 21: 394-407. https://doi.org/10.1111/j.1365-2435.2007.01283.x

Rebecca J. Fox, Jennifer M. Donelson, Celia Schunter, Timothy Ravasi, Juan D. Gaitán-Espitia; Beyond buying time: the role of plasticity in phenotypic adaptation to rapid environmental change. Philos Trans R Soc Lond B Biol Sci 18 March 2019; 374 (1768): 20180174.

4) L. 82: the notion of facilitating ecological opportunity and how it is expressed on this figure should be elaborated in a clearer way, I would argue. Particularly the notion of 'biodiversity change potential' remains too vague to the reader. Moreover, is this figure and its conclusion valid worldwide, or biased to the most anthropogenic areas of the world including (NW)-Europe? This needs some treatment as well, I believe.

Response: Thank you for this helpful comment. We agree that the figure requires clearer explanation, particularly regarding Biodiversity Change Potential (BCP) and the spatial scope of the framework. We have revised the manuscript text concerning Condition 4 and the figure legend to clarify that BCP refers to the capacity of populations or communities to convert ecological opportunity into realized biodiversity change. We also clarify that the figure is a conceptual representation of timescale mismatch, not a claim that all regions of the world experience identical dynamics. The compression of ecological opportunity is expected to be strongest in highly modified, rapidly changing, or disturbance-prone systems, but the framework applies more broadly because different regions vary in the rate, intensity, and predictability of anthropogenic change. We have revised the text to make this spatial contingency explicit.

Revised text reads: "Condition 4 Biodiversity change potential: Even sustained opportunity and stable selection regimes cannot yield diversification without sufficient genetic and phenotypic variation, i.e. response capacity. We refer to this capacity as Biodiversity Change Potential (BCP): the availability of genetic, phenotypic, demographic, and ecological variation that allows populations and communities to convert niche

availability and niche discordance into realized change. At the evolutionary level, BCP depends on standing genetic variation, mutation, recombination, gene flow, and admixture. At the ecological level, it is shaped by dispersal, demographic recovery, species sorting, phenotypic plasticity, and microbiome-mediated functional shifts."

Revised figure legend reads: "Figure 1. Timescale mismatch constrains ecological opportunity in the Anthropocene. Ecological opportunity requires temporal overlap among four conditions: niche availability, niche discordance, selection consistency, and biodiversity change potential. Green indicates conditions that persist long enough to support biodiversity change; red indicates transient conditions; gray indicates conditions that have already disappeared before they can be acted upon. Biodiversity change potential refers to the capacity of populations or communities to convert ecological opportunity into realized biodiversity change through standing genetic variation, plasticity, dispersal, demographic recovery, species sorting, and microbiome-mediated functional shifts. Under relatively stable Holocene conditions, these components were more likely to persist over ecological and evolutionary timescales. Under Anthropocene conditions, long-term directional trends are increasingly superimposed on short-term extremes, habitat fragmentation, interaction rewiring, and recurrent disturbance, shortening the temporal overlap among these components. The figure is conceptual and does not imply uniform global dynamics; opportunity compression is expected to vary geographically and to be strongest in systems where anthropogenic change is rapid, recurrent, and spatially pervasive."

5) L. 89: 'days or years' need some further underpinning (clear examples/references) and of course there are quite a lot of organisms with short life cycles relative to our time perspective, think about several insects and other invertebrates. So, here some more elaboration to make the point more convincing would be useful.

Response: Thank you for this helpful clarification. We have revised the text to make clear that the relevant timescale is organism-specific rather than defined by absolute time.

Revised text reads: "Yet in the Anthropocene, niche space may open and close within days or years—faster than most organisms can respond. These absolute timescales hold very different consequences for different organisms: a transient window may span many generations for microbes or short-lived invertebrates, but less than one or only a few generations for long-lived organisms such as vertebrates or plants."

6) L. 99: '...faster than selection can act.' Add some words why this is the case.

Response: We have revised the sentence accordingly.

Revised text reads: "Environmental variability (Calvin et al, 2023; Laurance et al, 2014; Coumou & Rahmstorf, 2012; Haddad et al, 2015)—heatwaves, flooding, fragmentation—

can shift optimal traits across days to years, often faster than selection can act because adaptive change requires selection of traits across reproductive cycles."

7) L. 102-104: Please underpin this with references.

Response: Thank you. We have added references to support the idea that buffering processes can reduce the strength of selection, in line with our response to point 3) above.

We have added the following references:

GHALAMBOR, C.K., McKAY, J.K., CARROLL, S.P. and REZNICK, D.N. (2007), Adaptive versus non-adaptive phenotypic plasticity and the potential for contemporary adaptation in new environments. Functional Ecology, 21: 394-407. https://doi.org/10.1111/j.1365-2435.2007.01283.x

Rebecca J. Fox, Jennifer M. Donelson, Celia Schunter, Timothy Ravasi, Juan D. Gaitán-Espitia; Beyond buying time: the role of plasticity in phenotypic adaptation to rapid environmental change. Philos Trans R Soc Lond B Biol Sci 18 March 2019; 374 (1768): 20180174.

8) L. 109: Be precise what is meant by 'no consistent axis of selection'? Is this always by definition directional selection? I don't think so.

Response: We agree that "no consistent axis of selection" was imprecise and could be read as referring specifically to directional selection. Our intended point was broader: under fluctuating and spatially heterogeneous environmental change, the selective target itself can move, such that the phenotypes favored in one context may no longer be favored in the next. We have therefore revised the sentence to refer to shifting trait optima and moving selective targets rather than to a "consistent axis of selection."

Revised text reads: "When the selective optimum moves faster than populations can track, adaptive trajectories fracture or stall (Salinas et al, 2019), leaving populations locked in maladaptation, persistence without diversification, or collapse."

9) L. 121: Add reference or phrase it as a prediction/working hypothesis rather than a certitude per se.

Response: We have revised the sentence to distinguish the referenced observation from the prediction emerging from our framework.

Revised text reads: "Many systems show evidence of shifts towards more generalist strategies (MacNeil et al, 2025; Rocha et al, 2017; Peters et al, 2022). We predict that this convergence reduces the ecological and genetic heterogeneity needed for diversification to occur."

10) L. 127: How would you define 'relative sable' here? The reader needs to have some assistance here.

Response: Thank you. We refer to stable relative to the timescale required for ecological sorting and evolutionary response. This is different for different organisms. We have revised the text to provide further detail.

Revised text reads: "Previously, niche availability was often sufficiently stable relative to organismal generation times, dispersal, and demographic recovery for ecological sorting and adaptive divergence to unfold. Under Anthropocene conditions, however, niche availability has become highly dynamic and transient."

11) L. 137: I am confused about the use of reference 46 as it refers to beneficial effects of invasive/exotic species, if I understood correctly.

Response: Thank you catching this. Indeed, this is a misplaced citation. We have replaced it with the following citation that also lends greater credence to the second part of the statement.

Sorte, C. J. B., Beshai, R A B, Dean, L S, R. J. Fales, E. L. Marin, and S. Sherzai. "Evolution and Invasive Species." In Encyclopedia of Evolutionary Biology, 282–87. Elsevier, 2016. https://doi.org/10.1016/b978-0-12-800049-6.00303-6.

12) L. 140: Could use a bit more elaboration to make your point.

Response: Thank you. We have elaborated this point to clarify the logical argument.

Revised text reads: "These mismatches generate transient ecological opportunities that tend to collapse before divergence. Ironically, the same mechanisms that create discordance can also buffer it. Thus, although invasions, microbial turnover, and community reassembly may lead to niche mismatches, compensatory responses through functional redundancy, altered resource access, or plasticity, can reduce fitness differences among genotypes and weaken the selective gradients required for adaptive divergence."

13) L. 149-150: Add reference or phrase it as a prediction/working hypothesis rather than a certitude per se.

Response: Thank you, changed accordingly.

Revised sentence reads: "Even when opportunity and selection align, we hypothesize that the potential for diversification is waning."

14) L. 151: I am not convinced that referring to evolutionary rescue only between brackets here is an optimal choice. Consider incorporating it more explicitly in the main text.

Response: We agree. “But see” makes evolutionary rescue look like a marginal caveat, but it is directly relevant.

Revised text reads: “Fragmentation (Haddad et al, 2015; Laurance et al, 2014), population decline (Dirzo et al, 2014; Hallmann et al, 2017; Bowler et al, 2019; Morgan et al, 2020), and genetic drift shrink the raw material on which selection can act. Evolutionary rescue can prevent extinction when adaptive variation is available and environmental change proceeds slowly enough for beneficial variants to spread (Lindsey et al, 2013; Bell & Gonzalez, 2009). However, under rapid or recurrent Anthropocene change, the same processes that erode population size, connectivity, and allelic diversity also reduce the probability that rescue can occur. The loss of allelic diversity constrains adaptive pathways, while strong directional pressures drive trait convergence toward generalist strategies.”

15) L. 158: Extinction debt likely is known by the majority of the readership, but I doubt whether it is also the case for 'facilitation debt'. Hence, I suggest adding a definition.

Response: We agree. ‘Facilitation debt’ is our analogous concept to ‘extinction debt’ and refers to the notion that positive interactions that could support recovery or diversification are delayed, unrealized, or not yet stabilized. We agree the term is not familiar. We have added definitions for *both* terms to the revised text.

Revised text reads: “Extinction debts are rising, with species persisting temporarily despite conditions that no longer support long-term survival. At the same time, facilitation debts may also accumulate, i.e. unrealized or delayed positive interactions that, if stabilized, could support recovery or rekindle diversification (Henriques & Osmond, 2020).”

16) L. 161-162: "Yet these arrangements are often unstable;...". Add reference or phrase it as a prediction/working hypothesis rather than a certitude per se.

Response: We have further expanded on this (following comment form the other reviewer), and we also provide a reference.

Revised text reads: “(...), yet these arrangements are often context-dependent. Competitive interactions can stabilize coexistence by limiting positive feedbacks and weakening effective interaction strengths, particularly in microbial communities (Coyte et al, 2015). However, when rewiring increases interaction turnover, asymmetric competitive dominance, predation, parasitism, or shifts in fitness rankings, it can accelerate ecological turnover and further destabilize selection (Bøhn et al, 2008).”

17) L. 168-169: That issue needs a more carefully discussion as there is evidence since a decade that microbial diversity may be correlated with other biodiversity components (e.g. Hanski et al 2012. PNAS).

Response: We agree and have revised this. The revised text clarifies the various ways in which the microbiomes can alter host states and also incorporates the argument of heritability.

Revised text reads: "Microbial communities can reorganize on short ecological timescales, often within days, potentially buffering hosts against environmental stress (Ziegler et al, 2017; Gillingham et al, 2025), <u>but they may also deteriorate host condition</u> (Hanski et al, 2012). Yet this flexibility is <u>often</u> reversible and fragile under repeated disturbance. <u>Even when microbiome-associated states are passed to offspring, their persistence depends on transmission fidelity, vertical inheritance, host filtering, and environmental reinforcement (Baldassarre et al, 2022; Murphy et al, 2023; Hanski et al, 2012</u>). It <u>therefore</u> provides short-term persistence <u>more readily than</u> long-term adaptation."

18) L. 169-170: Add reference or phrase it as a prediction/working hypothesis rather than a certitude per se.

Response: References have been added. Please refer to our previous response for detail regarding changes to the manuscript.

19) L. 173: Add reference(s).

Response: We have added Fox et al. (2019) in support of this statement.

Rebecca J. Fox, Jennifer M. Donelson, Celia Schunter, Timothy Ravasi, Juan D. Gaitán-Espitia; Beyond buying time: the role of plasticity in phenotypic adaptation to rapid environmental change. Philos Trans R Soc Lond B Biol Sci 18 March 2019; 374 (1768): 20180174.

20) L. 174: I find it somewhat strange to make use of the different labels 'ecological' and 'microbial' as microbes also are involved in ecological dynamics.

Response: Agreed; although we would argue that it's not automatically on everyone's radar, which is why we wanted to emphasize it here.

Revised sentence reads: "As ecological <u>dynamics, including</u> microbial dynamics, interact, niche discordance becomes both amplified and transient."

21) L. 180: Add concisely why that is the case.

Response: Revised accordingly.

Revised sentence reads: "Selection consistency remains the most vulnerable condition under rapid change because shifting environmental pressures repeatedly alter which traits are favored."

22) L. 184: Add reference(s)

Response: We have added the below reference to support this statement.

Kolodny, Oren, and Hinrich Schulenburg. "Microbiome-Mediated Plasticity Directs Host Evolution along Several Distinct Time Scales." Philosophical Transactions of the Royal Society of London. Series B, Biological Sciences 375, no. 1808 (September 28, 2020): 20190589. https://doi.org/10.1098/rstb.2019.0589.

23) L. 192-194: Not clear to the reader as you indicate the buffering capacity on the one hand, but the fragile nature on the other. Needs elaboration to clearly inform the reader about this kind of paradox.

Response: We have revised the text to make this paradox explicit and to clarify that buffering capacity and fragility are two linked properties of mutualistic dependence.

Revised text reads: "Mutualistic networks—from plant–pollinator systems to host–microbe symbioses—can help maintain diversity when interaction partners remain available, functionally redundant, and temporally synchronized. Under these conditions, mutualisms can buffer small populations and preserve genetic variation. However, this buffering capacity also creates dependence: when key partners are lost, interaction timing shifts, or network structure breaks down, formerly stabilizing interactions can become sources of vulnerability. Such breakdowns can trigger extinction debts (Kuussaari et al, 2009), while network reassembly may take decades, falling out of sync with the pace of environmental change."

24) L. 206: 'migration'. In the scientific literature there is a confusing use of the term as you mean 'dispersal' here and not 'migration' as a behavioural ecologist would interpret it as a seasonal movement without gene flow consequences. I know as you discuss further also 'assistant migration' (L.224) (which again is about dispersal), I would suggest at least the first time you refer to migration to clarify that you refer by migration to dispersal (with emigration as key component here).

Response: Thank you for this important point. As correctly pointed out by you, we use the term in the population-genetic sense to refer to dispersal among populations. To avoid ambiguity, we have clarified the term at the instance here use "dispersal" where this better reflects the intended meaning.

Revised text reads: "Diversification potential hinges on the generation and maintenance of variation across scales. While mutation and recombination remain essential, ecological processes—such as migration (i.e., dispersal among populations, including emigration from a source population and successful establishment at a novel site, both of which can contribute to gene flow), demographic shifts, and species turnover—rapidly reshape the pool of available diversity (Uecker & Hermisson, 2016; Hermann & Becks, 2022; Ceballos et al, 2017)."

25) L. 229: Figure 2. I have some doubts about the use of 'ecological adjustment'; at least it needs a clear definition.

Response: Thank you. We meant it as a collective term to describe the sum of all processes that allows populations to persist temporarily, including shifts in community composition, species interactions, habitat use, or demographic structure. We agree it's a generic label and exchanged it with 'ecological reassembly'. We have also added a clarifying sentence in the figure legend.

Revised figure legend reads: "(...) Different biological response mechanisms operate across distinct temporal domains: microbiome-mediated flexibility and behavioral and physiological plasticity can respond rapidly to acute environmental shifts; ecological reassembly, here defined as non-genetic shifts in dispersal, community composition, species interactions, trophic structure, or habitat use, can support intermediate responses; and genetic adaptation and diversification require the longest periods of environmental consistency. (...)"

Dear Christian,

Congratulations on an excellent manuscript! I am very pleased to inform you that it has been accepted for publication in The EMBO Journal. Thank you for comprehensively addressing the initially raised referees' comments and concerns, and our editorial requests for changes and corrections.

Your manuscript will be processed for publication by EMBO Press. It will be copy edited and you will receive page proofs prior to publication. Please note that you will be contacted by Springer Nature Author Services to complete licensing information.

Should you be planning a Press Release on your article, please notify the production team via this form in order to coordinate publication and release dates: https://forms.cloud.microsoft/e/eH4A8JVP6g

If you have any questions, please do not hesitate to contact the Editorial Office. Thank you for your contribution to The EMBO Journal. Working with you has been a pleasure.

Best regards,

Ioannis

Ioannis Papaioannou, PhD
Editor, The EMBO Journal
i.papaioannou@embojournal.org

*****
Please note that it is The EMBO Journal policy for the transcript of the editorial process (containing referee reports and your response letters) to be published as an online supplement to each paper. If you should prefer removal of any referee-only figures included in the point-by-point response(s), e.g. because they may still be used for future publication or because they have been reproduced from published work by others, please do let us know immediately via response email.
More information is available here: https://link.springer.com/partners/embo-press/editorial-policies#Peer%20review

** Click here to be directed to your login page: https://emboj.msubmit.net
